# Enantio-alteration of gene transcription associated with bioconcentration in adult zebrafish (*Danio rerio*) exposed to chiral PCB149

**DOI:** 10.1038/srep19478

**Published:** 2016-01-20

**Authors:** Tingting Chai, Feng Cui, Pengqian Mu, Yang Yang, Nana Xu, Zhiqiang Yin, Qi Jia, Shuming Yang, Jing Qiu, Chengju Wang

**Affiliations:** 1 Institute of Quality Standards & Testing Technology for Agro-Products, Key Laboratory of Agro-product Quality and Safety, Chinese Academy of Agricultural Sciences; Key Laboratory of Agri-food Quality and Safety, Ministry of Agriculture, Beijing 100081, China; 2 College of Science, China Agricultural University, Beijing 100193, China

## Abstract

Enantioselective enrichment of chiral PCB149 (2,2’,3,4’,5’,6-hexachlorobiphenyl) was analysed in adult zebrafish (*Danio rerio*) exposed to the racemate, (−)-PCB149, and (+)-PCB149. Greater enrichment of (−)-PCB149 compared to (+) PCB149 was observed following 0.5 ng/L exposure; however, as the exposure time and concentration increased, racemic enrichment was observed in adult fish exposed to the racemate. No biotransformation between the two isomers was observed in fish exposed to single enantiomers. When zebrafish were exposed to different forms of chiral PCB149, enantioselective expression of genes associated with polychlorinated biphenyls (PCBs) was observed in brain and liver tissues and enantioselective correlations between bioconcentration and target gene expression levels were observed in brain and liver tissues. The strong positive correlations between expression levels of target genes (*alox5a* and *alox12)* and PCB149 bioconcentration suggest that prolonged exposure to the racemate of chiral PCB149 may result in inflammation-associated diseases. Prolonged exposure to (−)-PCB149 may also affect metabolic pathways such as dehydrogenation and methylation in the brain tissues of adult zebrafish. Hepatic expression levels of genes related to the antioxidant system were significantly negatively correlated with bioconcentration following exposure to (+)-PCB149.

Although the production of PCBs ceased in many countries in the late 1970s and their extensive use in the past remain serious environmental problems^1^. In addition, PCBs are inadvertently generated by certain industrial processes including the production of paint pigments^2^ and adhesives^3^. Thus simply banning manufacture is in insufficient to reduce exposure^4^. PCBs can threaten human health via their potential carcinogenic effects^5^. Adverse health effects such as neurological effects, in humans exposed to PCBs have been investigated in laboratory and epidemiological studies^6^. Nineteen PCBs congeners contain a chiral axis and exist as two stable isomers at ambient temperature^7^, and racemic PCBs produced via the PCB manufacturing process have been released into the environment^8^. Although enantioselective enrichments of chiral PCBs do not occur in abiotic processes, different biological and toxicological effects are observed when the individual enantiomers interact with chiral molecules such as enzymes and biological receptors^9^. Chirality is thus a critical consideration for accurate assessment of pollutant toxicity and effects.

Contaminants released from various sources and entered into aquatic environment, , resulting in toxicity in aquatic organisms^10^. Many water sources have already been polluted by PCBs at different levels^11^,^12^. Fish are a powerful vertebrate model system for assessing the health of aquatic environments; physiological changes in fish can serve as biomarkers of environmental pollution^14^. Although the transformation of PCB isomers is limited in fish, non-racemic PCBs have been observed in fish exposed to PCBs via water and diet^15^. Enantioselectivity of (−)-PCB84 and (+)-PCB132 have been observed when rainbow trout were exposed to a mixture of PCBs84, 132, and 174^16^, whereas no enantioselective enrichment has been observed in rainbow trout exposed to PCBs95, 132, 149, 174 and 183^17^. Non-racemate enrichment of PCBs has also been observed in arctic char *(Salvelinus alpinus)*^18^.

Zebrafish, an informative model for studies of human biology^19^,^20^, is a standard species in aquatic toxicology studies^21^; however, owing to the rapid life cycle of this species, the applications of zebrafish extend far beyond the fields of toxicology, cell biology, and developmental genetics^22^. Compared to mammals, fish exhibit far lower abundances and activities of CYPs, lower rates of electron transportation, and less active mono-oxygenates, resulting in far lower capacities for the detoxification and biotransformation of xenobiotic chemicals such as PCBs^23^. New molecular approaches, however, can provide greater insight into toxicological injury: changes in mRNA transcript levels are a key component of the biological response to exposure to a chemical contaminant^24^. Therefore, ecological screening methodologies based on genomics are potentially a powerful means of evaluating the effects of anthropogenic contaminants.

Among chiral PCBs, information on chiral PCB149 in biological samples is limited. The present study was therefore designed to investigate the enantioselective enrichment and expression of genes associated with PCBs exposure. This study also sought to assess the relationship between bioconcentrations and gene transcripts to further explore the potential pathogenic mechanisms of PCBs.

## Results

### Method validation

Satisfactory recovery of 97.4–103.6% with relative standard deviation of 2.3–10.1% were obtained at three spiked concentration levels (0.4 ng/L, 0.1 μg/L, 1 μg/L) in water. As shown in Table 1, the actual concentrations of chiral PCB149 were less than 20% of the theoretical concentrations for all test periods and no isomerization was observed for either of the two isomers in water. Therefore, the theoretical concentration appropriately represented the actual concentration during the exposure experiments.

Recovery estimations for adult zebrafish were performed with three spiked concentrations (10 μg/L, 250 μg/L, 5 mg/L) and mean recoveries of 99.7–100.4% with relative standard deviations of 5.3–10.6% were obtained. Recovery rates and their standard errors were thus considered acceptable.

### Non-racemic enrichment

**Figure 1:**
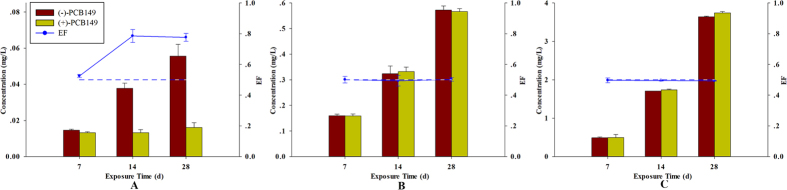
Concentrations of (−)-PCB149 and (+)-PCB149 and EF values following exposure to racemate. (**A**) adult zebrafish exposed to 0.5 ng/L; (**B**) adult zebrafish exposed to 0.1 μg/L; (**C**) adult zebrafish exposed to 0.25 μg/L. Asterisks denote significant differences between treatments and control (determined by Dunnett post hoc comparison, P < 0.01, **). Error bars indicate standard deviations.

Significant differences in enantiomer fraction (EF) values (EFs > 0.5) were observed at a dose concentration of 0.5 ng/L (Fig. 1), indicating preferential enrichment of (−)-PCB149 and consequently, enantioselective enrichment. By contrast, enantioselectivity was not observed at exposure of 0.1 μg/L and 0.25 μg/L. The concentrations of (−)/(+)-PCB149 were analysed and the data are presented in Table 2.

### Hepatic gene expression

**Figure 2:**
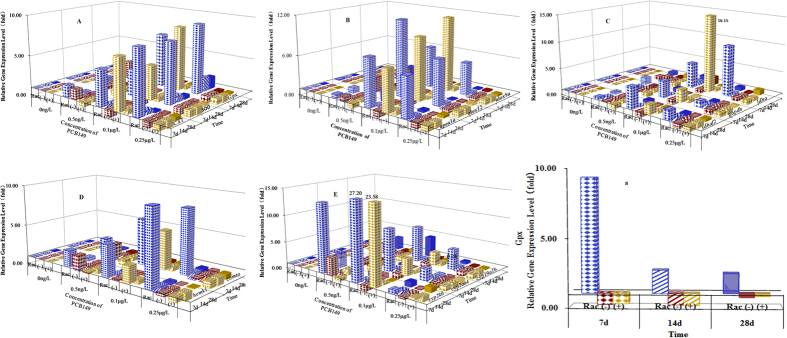
The effect of the duration and concentration of racemic-, (−)-, and (+)-PCB149 on hepatic expression levels of indicated genes relative to the no-addition control. (**A**) transcription of genes involved in antioxidant system; (**B**) transcription of genes involved in lipid-peroxidation; (**C**) transcription of genes involved in dehydrogenation; (**D**) transcription of genes involved in methylation; E: transcription of CYP450; (**a**) transcription of *Gpx* genes at 0.25 μg/L with time.

The transcription of genes related to the antioxidant system (*sod*, *cat*, and *Gpx;* Fig. 2A) increased significantly with increasing exposure concentrations at 7 days post exposure (dpe) for the PCB racemate. Upon prolonged exposure to the racemate, the expression levels of the three target antioxidant genes in liver tissue decreased at the three exposure concentrations, respectively. By contrast, the expression levels of this genes were not altered by exposure to either (−)-PCB149 or (+)-PCB149. The tendency towards upregulation of *Gpx* expression levels declined with prolonged exposure time to the racemate at 0.25 μg/L (Fig. 2a), but the tendency towards downregulation of *Gpx* expression levels was enhanced by prolonged exposure to (−)-PCB149 and (+)-PCB149 at 0.25 μg/L (Fig. 2a). Enantioselective hepatic expression was observed for exposure to 0.5 ng/L of each form of PCB149 at 7, 14, and 28 dpe.

At higher exposure concentrations (0.1 μg/L and 0.25 μg/L), clear changes in the expression of genes related to the lipid-peroxidative pathway (*apoa1a*, *alox12*, and *alox5a*) were observed in liver tissue (Fig. 2B). For 0.25 μg/L at 14 dpe, hepatic transcription of *apoa1a*, *alox12*, and *alox5a* tended to be normal (similar to β-actin expression). No enantioselective expression of the three genes was observed in fish exposed to 0.1 μg/L (−)-PCB149 or (+)-PCB149.

Hepatic transcription of genes related to the dehydrogenase system (*sdhaf2*, *gapdh*, and *ldha*) (Fig. 2C) was clearly enantioselective for exposure to 0.5 ng/L at 7, 14, and 28 dpe: the fold inhibition of *sdhaf2* and *gapdh* expression increased to 20-fold at 0.5 ng/L and to 25-fold at 0.25 μg/L at 14 dpe for the racemate. Downregulation of *sdhaf2* and *ldha* was observed when (−)-PCB149 and (+)-PCB149 were present separately at 0.25 μg/L at 7 dpe; however, upregulation was observed when both isomers were present.

Exposure to increasing concentrations of the PCB racemate resulted in increased expression levels of *hemk1* and *comta*, genes relating to methylation in liver tissues (Fig. 2D) at 7 dpe for the racemate. By contrast, for (−)-PCB149, decreases in *hemk1* and *comta* expression were observed at increasing exposure concentrations at 7 dpe. Enantioselectivity was also observed at 7 dpe. Hepatic transcription (Fig. 2E) of genes related to hydroxylation (*cyp2k6*, *cyp2aa4*, and *cyp19a1b)* in an enantioselective manner was observed under certain exposure conditions; for example, the hepatic expression of *cyp19a1b* increased by exposure to 0.1 μg/L of all three forms at 7 dpe.

### Cerebral gene expression

**Figure 3:**
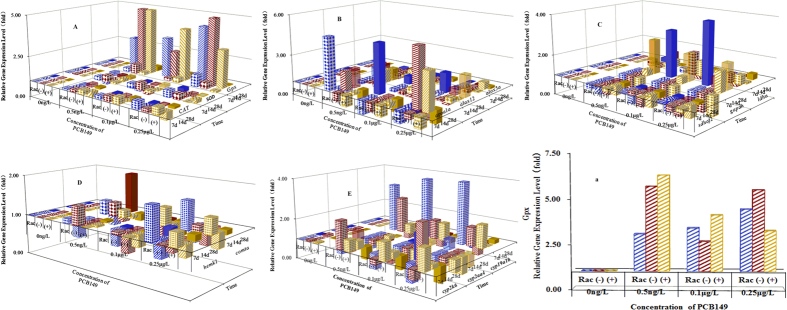
The effect of the duration and concentration of racemic-, (−)-, and (+)-PCB149 on cerebral expression levels of indicated genes relative to the no-addition control. (**A**) transcription of genes involved in antioxidant system; (**B**) transcription of genes involved in lipid-peroxidation; (**C**) transcription of genes involved in dehydrogenation; (**D**) transcription of genes involved in methylation; (**E**) transcription of CYP450; (**a**) transcription of *Gpx* genes after 14 days of exposure at different concentrations.

Small fluctuations in the levels of the three antioxidant genes were observed in brain tissues (Fig. 3A), with the exception of *Gpx* at 14 dpe (Fig. 3a). The expression levels of *Gpx* expression increased with exposure to increasing concentrations of the racemate and cerebral transcription of *Gpx* increased, while for (+)-PCB149, cerebral transportation of *Gpx* decreased with increasing exposure concentration.

Significant differences in the expression levels of three lipid-peroxidative pathway-related genes were observed in (−)-PCB149 and (+)-PCB149-treated fish. Downregulation of *apoa1a*, upregulation of *alox12*, and near-normal expression of *alox5a* were observed in the brain tissue of fish exposed to 0.25 μg/L of all three forms at 14 dpe (Fig. 3B).

No fluctuations in *ldha* expression were observed in brain tissue (Fig. 3C) during the exposure period; however, with increased exposure time, the initial downregulation of gapdh expression was followed by upregulation for each form of PCB149 at all three concentrations of each form.

Small fluctuations in the cerebral transcription levels of *hemk1* and *comta* (Fig. 3D) were observed and cerebral expression of *cyp2k6* was inhibited at 7 dpe for the racemate (Fig. 3E). Exposure to increasing concentrations of (−)-PCB149 resulted in a decrease in the cerebral expression of *cyp2k6*, whereas exposure to (+)-PCB149 resulted in enhanced expression of *cyp2k6*. Significant downregulation of *cyp2aa4* and up-regulation of *cyp19a1b* were observed at 7 dpe. The expression of *cyp19a1b* in brain tissue differed depending on the exposure time and concentration. At increasing concentrations of all three forms, for example, *cyp19a1b* expression was either enhanced (7 dpe) or inhibited (14 dpe and 28 dpe). Moreover, at the same concentration, cerebral transcription of *cyp19a1b* decreased with prolonged exposure time.

### Chemometrics

**Figure 4:**
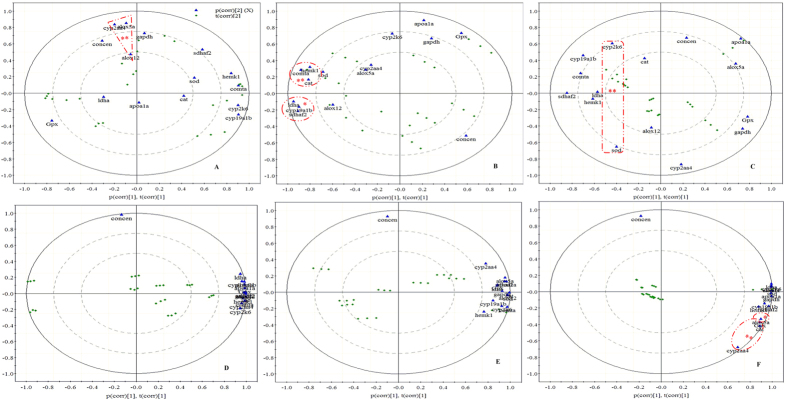
Loading diagram for PCA analysis of PCB149 concentrations and relative transcriptional level of indicated genes after exposure to: (**A,D**) racemate; (**B,E**) (−)-PCB149; (**C,F**) (+)-PCB149; (**A–C**) in brain; (**D–F**) in liver. Green diamond marks represent samples and blue triangle marks represent genes. *P < 0.05 according to Spearman’s test; **P < 0.01 according to Spearman’s test.

To assess enantioselective enrichment, EF values were calculated using bioconcentration data. To better understand the relationship between concentration levels and fluctuations in the expression of target genes with various functions in liver and brain tissues, we performed correlation analyses (Table 3) and principal component analysis (PCA) on the loading Bi plots (Fig. 4).

## Discussion

Racemic PCB149 was previously detected in groupers (*Epinephelus marginatus*) collected between 1994 and 1995 from the northwest African Atlantic Ocean^25^ as well as in Arctic cod (*Boreogadus saida*)^26^. The results of the exposure experiments at higher concentration of PCB149 described here are consistent with these reports. An enantioselective capacity for chiral PCB bioprocessing has also been observed in Lake Superior trout^27^. Fish species are considered to have low PCB biotransformation capacities^28^ and in the present study, no biotransformation between the two isomers was observed.

Interestingly, we previously demostrated that embryo-larvae have a higher accumulation capacity for PCB149 than adult zebrafish: at the same exposure concentration (0.1 μg/L), the concentration of (+)-PCB149 in embryo-larvae reached 3.69 mg/L at 11 dpe, whereas the concentration of (+)-PCB149 reached only 0.57 mg/L after 28 dpe in adult zebrafish. This difference may be due to the ability of PCB149 to permeate the embryonic membrane^29^; the chorion is composed of lipid and thus may facilitate the passage of lipophilic PCB149 into the embryo. During the endogenous feeding period, nutrition is provided by the yolk sac before the yolk sac completely disappears. Endogenous feeding and a fully developed metabolic pathway^30^ thus may also enhance PCB149 bioaccumulation in larvae. However, PCB149 mainly enters adult zebrafish tissues through gill respiration and the skin, which may result in slower bioaccumulation.

PCAs analysis on loading Bi plots (Fig. 4) was performed to investigate the relation between target genes with different functions and bioconcentration. As shown in Fig. 4A–C, expression of the target genes in brain tissues exhibited a relatively scattered trend, whereas the expression levels of the target genes in liver tissues were relatively concentrated in Fig. 4D–F. The PCAs plots also suggested that the changes in the expression levels of the target genes with different functions were more closely correlated with bioconcentration in liver tissues than in brain issues. This finding was confirmed by the correlation analyses (Table 3).

Enantioselective correlations between bioconcentration and the target genes were observed in the brain and liver tissues of adult zebrafish exposed to different forms of chiral PCB149. The correlation coefficient and PCA results indicated that the cerebral expression levels of *alox5a*, *alox12*, and *cyp2aa4* were significantly positively correlated with bioconcentration following zebrafish exposure to the racemate. In zebrafish exposed to (−)-PCB149, the cerebral expression of *cyp2aa4, comta*, and *hemk1* was significantly negatively correlated with bioconcentration, and *sdhaf2, ldha*, and *cyp19a1b* expression was highly negatively correlated with bioconcentration. The cerebral expression of *sod* and *cyp2k6* was significantly negatively correlated with bioconcentration following exposure to (+)-PCB149. The bioactive compounds generated by genes related to the lipoxygenase pathway may result in a variety of biological activities important for carcinogenesis^31^. Arachidonate lipoxgenase (alox) enzymes metabolize arachidonic acid and thus generate potent inflammatory mediators, thereby leading to inflammation-associated diseases^32^. Both alox5a and alox12 enzymes have been described as pro-carcinogenic: For example, the alox12 enzymes may increase the expression of genes encoding proinflammatory cytokines such as tumour necrosis factors^33^ and aberrant expression of *alox5* has been reported in patients with breast cancer^34^. We observed highly positive correlations between the expression of these genes (*alox5a*, *alox12)* and PCB149 bioconcentration, suggesting that prolonged exposure to the racemate of PCB149 may result in inflammation-associated diseases.

Furthermore, prolonged exposure to (−)-PCB149 may affect metabolic pathways in the brain tissue of adult zebrafish. The catechol-*O*-methyltransferase (COMT) enzyme, which forms reactive adducts with DNA, is considered to be a potentially important contributor to cancer development. Garner and colleagues have determined that polymorphic variants of the COMT gene are associated with an increased risk of hormone-related cancer^35^. Succinate dehydrogenase (SDH), which is complex II of the respiratory chain, is situated at the centre of two essential energy-producing cell processes: the tricarboxylic acid cycle and mitochondrial oxidative phosphorylation. Decreased SDH levels may result in a limited capacity for oxidative phosphorylation and consequently ATP production^36^ and *sdhaf2* plays a role in hereditary disease^37^. The LDHA enzyme, a metabolic enzyme in the glycolysis pathway, plays a critical role in cancer progression^38^ and dysregulation of LDHA expression has been observed in various cancer cell types including squamous cell carcinoma^39^ and breast cancer^40^. The negative correlations between bioconcentration and the expression of these genes in brain tissue of zebrafish exposed to (−)-PCB149 observed in the present study suggest that prolonged exposure to (−)-PCB149 could cause related cancer.

No obvious correlation between PCB149 bioconcentrations and the changes in the hepatic expression levels of target genes were observed in zebrafish exposed to the racemate and (−)-PCB149; however, the hepatic expression levels of *cat, Gpx*, and *cyp2aa4* exhibited significant negative correlations with bioconcentration for (+)-PCB149 exposure. Oxidative stress occurs when there is an imbalance in the biological oxidant-to-antioxidant ratio, which resulted in oxidative damage to lipids, proteins, carbohydrates, and nucleic acids^41^. In fish, this oxidative stress in a defensive mechanism involving the activation of the antioxidant system to detoxify reactive oxygen species (ROS)^42^. The enzyme SOD converts ROS to H_2_O_2_ and the enzymes CAT and Gpx then catalyse the transformation of H_2_O_2_ to oxygen and water^43^. In this study, adult zebrafish exposed to (+)-PCB149 exhibited high negative correlations between the cerebral expression levels of *sod* and PCB bioconcentration as well as between the hepatic expression levels of *cat* and *Gpx* genes and PCB concentration, suggesting that increases in (+)-PCB149 bioconcentrations may result in change in the expression levels of *cat* and *Gpx* in liver tissue following changes in the expression levels of *sod* in brain tissue.

The P450 enzymes typically catalyse mono-oxygenase reactions that involve oxygen molecular and an equivalent number of electrons. These enzymes are encoded by different members of the CYP gene superfamily^44^. The *CYP2* gene family in zebrafish is a novel subfamily with no orthology to human and mammalian *CYP* genes^45^. We observed different correlations between the expression levels of *cyp2aa4* and PCB149 bioconcentration: a significant positive correlation was observed following exposure to the racemate, whereas a significant negative correlation with cerebral gene expression levels was observed following exposure to (−)-PCB149. A significant negative correlation was also observed for hepatic gene expression levels in liver tissue following (−)-PCB149 exposure. However, the regulation and function of CYP2s remains poorly understood. CYP19, known as aromatase, catalyses the final step in the conversion of androgens to oestrogens^44^. *Cyp19a1b* (generally named *cyp19b*) is primarily expressed in brain tissue, and brain *cyp19a1b* is physiologically significant for oestrogen-dependent neurogenesis^46^ and the regulation of the gonadal-pituitary-hypothalamic axis^47^. We did not observe correlation between the hepatic expression levels of *cyp19a1b* and bioconcentration; onlyfor (−)-PCB149 exposure did expression levels of *cyp19a1b* in the brain exhibit a strong negative correlation with bioconcentration.

## Method

### Zebrafish husbandry

Juvenile AB strain zebrafish (*Danio rerio*) were obtained from Beijing Hongdagaofeng Aquarium Department and cultured in a fish facility (Esen Corp.) at 26 °C with a photoperiod of 14/10 (light/dark)^31^. Six-month-old adult zebrafish were selected for this study and were fed dried brine shrimp (equivalent to 2% of the fish body weight) daily.

### Chemicals and reagents

Racemic PCB149 (99.9%) was provided by Dr. Ehrenstorfer GmbH (Germany). The racemate was separated and prepared on a Lux Cellulose-2 column (250 × 4.6 mm, 5 μm, Phenomenex, Torrance, CA) using an Agilent 1200 series high performance liquid chromatography (HPLC) instrument (Wilmington, DE) with 100% *n*-hexane as the mobile phase at a flow rate of 1.0 mL/min. The enantiomers (−)-PCB149 and (+)-PCB149^48^ were repeatedly collected separately, concentrated to dryness using a nitrogen-evaporator (Hangzhou Allsheng Instruments company, China) and then dissolved in acetone (Fisher). The purities and concentrations of the isomers were determined by gas chromatography-mass spectrometry (GC-MS).

An Agilent 7890A/5975C GC-MS system equipped with a Chirasil-Dex capillary column (25 m × 0.25 mm; I.D. 0.25 μm df) from Agilent was used for purity and concentration determinations, and the oven temperature was programmed as follows: 60 °C for 2 min, 60–150 °C at 10 °C·min^−1^ (held for 5 min), 150–180 °C at 1 °C·min^−1^ (held for 22 min). The SIM ions were m/z 360 (quantification ion), 362, and 358^7^. The purities of (−)-PCB149 and (+)-PCB149 were higher than >98.0%.

### Exposure and sample collection

This study was performed in accordance with Chinese legislation and was approved by the independent animal ethics committee at China Agricultural University. Adult zebrafish were exposed to racemate, (−)-PCB149, and (+)-PCB149 at 0.5 ng/L, 0.1 μg/L, and 0.25 μg/L plus solvent control. Three tanks were used for each exposure concentration of each target compound; one tank represented one repetition. A total of 40 six-month-old adult fish were assigned at random to one tank (25 L) containing 20 L test solution was assigned to randomly contain forty six-month-old adult fish. During the test, 50% of test solution was changed every day until the end of experiment at 28 days. At 7, 14, and 28 days post exposure (dpe), twelve adult fish from one tank were randomly selected and assigned to two groups: six for gene expression analysis and six for bioconcentration analysis. For analysis, the fish were anesthetized with MS-222 on ice. Liver and brain tissues were obtained by dissection, and stored overnight in RNA storage solvent at 4 °C before being removed from the RNA storage solvent and stored at −80 °C until RNA extraction. During the exposure experiments described above, the exposure environment including temperature, humidity, and light cycle was identical to the culture environment.

### Quantification of PCB149 levels in water and biological samples

For exposure analysis, the tank water was sampled for GC-MS analysis at five time points: 0, 1, 7, 14, and 28 dpe. Water samples (1 L) were subjected to five liquid-liquid extractions with *n*-hexane (100 mL each time) in a separatory funnel with violent shaking. The *n*-hexane layer was transferred to heart-shaped flasks and concentrated to near-dryness by rotary evaporation (Shanghai Ailang Instruments, Shanghai, China) at 35 °C. The concentrated solutions were then blown to dryness by nitrogen evaporation and the residue was again dissolved in 0.1 mL of isooctane for GC-MS analysis. Six adult zebrafish weighed for follow-up in a previous study^49^ and were also processed for GC-MS analysis.

### Gene expression studies

Total RNA was extracted from liver/brain tissue using an RNAprep Pure Tissue Kit (Tiangen Biotech, China). The quality of the isolated RNA was evaluated based on the quality of the 28 s and 18 s rRNAs by 2% agarose gel electrophoresis and the purity of the RNA preparations was assessed by OD_260_/OD_280_ ratio. The concentration of the RNA was determined by OD_260_ using a UV1240 spectrophotometer (Perkin Elmer, USA). First-strand complementary DNA (cDNA) was synthesized from 0.5 μg of total RNA using a FastQuant RT Kit (Tiangen Biotech).

Quantitative real-time polymerase chain reaction (qPCR) was performed using a SuperReal PreMix Plus Kit (Tiangen Biotech) and an ABI 7500 qPCR system (Applied Biosystems, USA). Primers were designed using the Primer 6.0 tool and are shown in Table 4. The housekeeping gene β-actin was used as an internal standard to eliminate variations in mRNA and cDNA quantity and quality. Three-step qPCR was used to quantify the relative levels of housekeeping and target genes: 95 °C for 15 min; 40 cycles of 95 °C for 10 s, 60 °C for 20 s, and 72 °C for 32 s. A melting curve analysis was also performed to confirm the specificity of PCR product as a single peak. Relative quantification of target genes normalized to β-actin levels was performed by the 2^−ΔΔCt^ method.

### Statistical analysis

EFs were evaluated in samples to determine enantiomeric compositions^50^; the EFs was defined as the enantiomeric concentration ratio (−)/[(−)+(+)] for PCB149. EF values were in the range of 0–1, where 0.5 represents a racemate.

Statistical analyses were performed using SPSS16.0 software. Differences were determined by one-way ANOVA followed by post hoc Dunnett tests. All data are expressed as mean ± standard error of the mean and differences with P < 0.05 were considered statistically significant. Relationships between bioconcentrations and target genes transcription levels were analysed by PCA, followed by the calculation of correlation coefficients to further confirm these relationships.

## Additional Information

**How to cite this article**: Chai, T. *et al.* Enantio-alteration of gene transcription associated with bioconcentration in adult zebrafish (*Danio rerio*) exposed to chiral PCB149. *Sci. Rep.* **6**, 19478; doi: 10.1038/srep19478 (2016).

## Figures and Tables

**Table 1:** The detected PCB149 concentrations in collected water samples.

Test period	Theoretical concentration	Actual concentration		
		*Racemic exposure*^a^	(−)-PCB149 exposure	(+)-PCB149 exposure
0 dpe	control	–	–	–
	0.5 ng/L	0.55 ± 0.05 ng/L	0.53 ± 0.03 ng/L	0.49 ± 0.07 ng/L
	0.1 μg/L	0.11 ± 0.01 μg/L	0.10 ± 0.00 μg/L	0.09 ± 0.00 μg/L
	0.25 μg/L	0.27 ± 0.04 μg/L	0.25 ± 0.01 μg/L	0.23 ± 0.05 μg/L
1 dpe	control	–	–	–
	0.5 ng/L	0.45 ± 0.03 ng/L	0.43 ± 0.02 ng/L	0.40 ± 0.06 ng/L
	0.1 μg/L	0.11 ± 0.00 μg/L	0.09 ± 0.01 μg/L	0.10 ± 0.01 μg/L
	0.25 μg/L	0.22 ± 0.02 μg/L	0.24 ± 0.00 μg/L	0.21 ± 0.03 μg/L
7 dpe	control	–	–	–
	0.5 ng/L	0.52 ± 0.04 ng/L	0.45 ± 0.04 ng/L	0.51 ± 0.05 ng/L
	0.1 μg/L	0.10 ± 0.01 μg/L	0.10 ± 0.00 μg/L	0.11 ± 0.01 μg/L
	0.25 μg/L	0.26 ± 0.01 μg/L	0.27 ± 0.00 μg/L	0.24 ± 0.00 μg/L
14 dpe	control	–	–	–
	0.5 ng/L	0.49 ± 0.01 ng/L	0.55 ± 0.02 ng/L	0.52 ± 0.06 ng/L
	0.1 μg/L	0.12 ± 0.00 μg/L	0.10 ± 0.00 μg/L	0.09 ± 0.01 μg/L
	0.25 μg/L	0.22 ± 0.04 μg/L	0.27 ± 0.01 μg/L	0.24 ± 0.03 μg/L
28 dpe	control	–	–	–
	0.5 ng/L	0.51 ± 0.07 ng/L	0.48 ± 0.05 ng/L	0.53 ± 0.04 ng/L
	0.1 μg/L	0.11 ± 0.00 μg/L	0.10 ± 0.01 μg/L	0.11 ± 0.00 μg/L
	0.25 μg/L	0.26 ± 0.00 μg/L	0.27 ± 0.01 μg/L	0.23 ± 0.02 μg/L

^a^This concentration is the total contamination of two isomers.

**Table 2:**
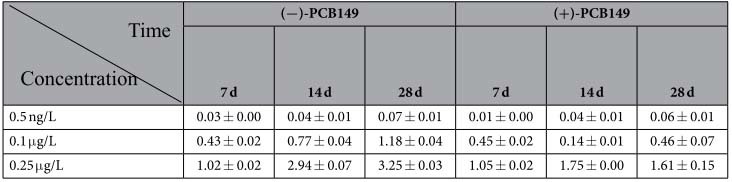
Concentrations of (−)-PCB149/(+)-PCB149 in adult zebrafish when exposed to enantiomers.

**Table 3:** Correlation coefficients between bioconcentration and expression levels of all target genes.

Gene	Correlation coefficient					
	Brain			Liver		
	Racemate	(−)-PCB149	(+)-PCB149	Racemate	(−)-PCB149	(+)-PCB149
*sod*	0.057	−0.331	−0.675**	−0.351	−0.095	−0.331
*cat*	−0.141	−0.496**	−0.032	−0.336	0.002	−0.520**
*Gpx*	0.080	−0.323	−0.289	−0.187	−0.122	−0.522**
*sdhaf2*	0.378	−0.394*	0.088	−0.217	−0.042	−0.454*
*ldha*	−0.05	−0.435*	−0.113	0.161	0.140	−0.087
*gapdh*	0.119	−0.112	0.125	−0.270	−0.176	−0.321
*apoa1a*	−0.231	−0.319	−0.016	0.004	−0.087	−0.330
*alox5a*	0.543**	−0.002	−0.147	−0.038	0.152	−0.513*
*alox12*	0.394**	0.082	−0.1	−0.210	0.016	−0.178
*comta*	−0.021	−0.500**	−0.05	−0.351	−0.041	−0.362
*hemk1*	0.040	−0.574**	−0.153	−0.266	−0.028	−0.174
*cyp2k6*	−0.313	−0.217	−0.736**	−0.340	−0.036	−0.239
*cyp2aa4*	0.639**	−0.006	−0.267	−0.226	0.070	−0.502**
*cyp19a1b*	−0.238	−0.437*	0.210	−0.019	0.013	−0.322

*P < 0.05 according to Spearman’s test;

**P < 0.01 according to Spearman’s test.

**Table 4:** Sequences of primer pairs used for real-time quantitative PCR.

Target Gene	Full name	Primer Sequences	Accession Number
*β-actin*	Beta-actin	F: 5′-TGGACTCTGGTGATGGTGTGAC-3′	AF057040.1
		R: 5′-GAGGAAGAAGAGGCAGCGGTTC-3′	
*sod*	Cu/Zn-superoxide dismutase	F: 5′-GTCGTCTGGCTTGTGGAGTG-3′	Y12236
		R: 5′-TGTCAGCGGGCTAGTGCTT-3′	
*cat*	Catalase	F: 5′-AGGGCAACTGGGATCTTACA-3′	AF170069
		R: 5′-TTTATGGGACCAGACCTTGG-3′	
*Gpx*	Glutathione peroxidase	F: 5′-AGATGTCATTCCTGCACACG-3′	AW232474
		R: 5′-AAGGAGAAGCTTCCTCAGCC-3′	
*apoa1a*	Apolipoprotein A-la	F: 5′-TGACAACCTGGACGGAACCGACTA-3′	NM131128
		R: 5′-GCTGCTTGGTGTTCTCCATCAACTG-3′	
*alox12*	Arachidonate 12-lipoxygenase	F: 5′-CGATCTTCACCAGCACAGCACAACA-3′	NM199618
		R: 5′-TGTCAGGCAGCGTGTCCATAATCAT-3′	
*alox5a*	Arachidonate 5-lipoxygenase a	F: 5′-CGAGAGAGGAGCGGTGGACTCATAT-3′	NM001256747
		R: 5′-GTCATCAACCAACCAGCGGAAGCA-3′	
*sdhaf2*	Succinate dehydrogenase complex assembly factor 2	F: 5′-TGCTCCAGAACCGACCATCCTTGA-3′	NM001082864
		R: 5′-TGCGGCTCTCGTACAGCAGTCT-3′	
*gapdh*	Glyceraldehyde-3-phosphate dehydrogenase	F: 5′-GACGCTGGTGCTGGTATTGCTCTC-3′	NM001115114
		R: 5′-CCATCAGGTCACATACACGGTTGCT-3′	
*ldha*	Lactate dehydrogenase A4	F: 5′-TGCTCGTTTCCGCTACTTGATGGG-3′	NM131246
		R: 5′-ACGCTCTTCCAGTCCTCCTTGTCTT-3′	
*hemk1*	HemK methyltransferase family member 1	F: 5′-TGCGGTTGTTGTGCTGTGGTAGT-3′	NM001114419
		R: 5′-GATGCGGTGCAGGCTGAAGTGT-3′	
*comta*	Catechol-O-methyltransferase a	F: 5′-TGTTGGCATCTGTCCTGGTACTCCT-3′	NM001030157
		R: 5′-CGCTGTGGTCGTGATAGTCCTGTG-3′	
*cyp2aa4*	Cytochrome P450, family 2, subfamily AA, polypeptide 4	F: 5′-GCATCGTGGGTATAGTCCGCTATCC-3′	NM001002092
		R: 5′-CGCTCAACGGCTGTGCTGTTATTG-3′	
*cyp2k6*	Cytochrome P450, family 2, subfamily K, polypeptide 6	F: 5′-ACGCAGGGTTTGCATTGGAGAGAG-3′	NM200509
		R: 5′-CAGTTGGTGTGGCTTCGGATTCAGT-3′	
*cyp19a1b*	Cytochrome P450, family 19, subfamily A, polypeptide 1b	F: 5′-TCCGCTGTGTACCATGTCCTGAAGA-3′	NM131642
		R: 5′-CTGACTTCTGGAGACCTGGACCTGT-3′	
